# Engineering Properties and Correlation Analysis of Fiber Cementitious Materials

**DOI:** 10.3390/ma7117423

**Published:** 2014-11-20

**Authors:** Wei-Ting Lin, Yuan-Chieh Wu, An Cheng, Sao-Jeng Chao, Hui-Mi Hsu

**Affiliations:** 1 Institute of Nuclear Energy Research, Atomic Energy Council, Executive Yuan, Taoyuan 325, Taiwan; E-Mails: wtlin@niu.edu.tw (W.-T.L.); ycwu@iner.gov.tw (Y.-C.W.); 2 Department of Civil Engineering, National Ilan University, 1 Shen-Lung Road, Ilan 260, Taiwan; E-Mails: chao@niu.edu.tw (S.-J.C.); hmhsu@niu.edu.tw (H.-M.H.)

**Keywords:** fiber cementitious materials, direct tensile strength, drop-weight test, abrasion test

## Abstract

This study focuses on the effect of the amount of silica fume addition and volume fraction of steel fiber on the engineering properties of cementitious materials. Test variables include dosage of silica fume (5% and 10%), water/cement ratio (0.35 and 0.55) and steel fiber dosage (0.5%, 1.0% and 2.0%). The experimental results included: compressive strength, direct tensile strength, splitting tensile strength, surface abrasion and drop-weight test, which were collected to carry out the analysis of variance to realize the relevancy and significance between material parameters and those mechanical properties. Test results illustrate that the splitting tensile strength, direct tensile strength, strain capacity and ability of crack-arresting increase with increasing steel fiber and silica fume dosages, as well as the optimum mixture of the fiber cementitious materials is 5% replacement silica fume and 2% fiber dosage. In addition, the Pearson correlation coefficient was conducted to evaluate the influence of the material variables and corresponds to the experiment result.

## 1. Introduction

Cementitious materials have long been used for civil infrastructure such as highways, bridges and buildings. However, cementitious materials inherit low tensile strength, are brittle, and have other shortcomings; and the unexpected deterioration of civil infrastructure has led to the improvement of durability caused structural damage, and repair costs have increased annually in the United States [1]. Studies focusing on lowering the cause of the durability degradation mechanism and exploring influential factors have been extensive. Based on their results, these studies proposed to increase the durability of the structures. However, the indicator of durability has not yet been clearly defined. Thus, establishing objective indicators of durability for proper assessment is necessary to ensure a sustainable design or maintenance [2,3]. Traditionally, the composition of cementitious materials includes cement, pozzolan, water, aggregate and or admixtures. Fiber has also been added in the fiber cementitious materials (FCM) since 1960 to improve concrete properties, particularly tensile strength, surface abrasion resistance and energy absorbing capacity [4,5,6]. The presence of fiber in cementitious materials helps to refrain crack growth and transfer load to the uncracked parts [7,8,9]. The specimen containing fibers has much higher ductility than the specimen without fibers. Fibers by bridging the cracks in the cementitious composites increase the energy absorption or toughness [10]. However, the properties of FCM would be affected by the volume fraction and aspect ratio of fiber. Lower fiber volume fraction is usually preferred by considering the material cost and workability [11]. According to the results of previous researches, the volume fraction was suggested up to 2% to obtain fiber effectiveness in cementitious materials [12].

It was also reported that combination of silica fume with steel fibers effectively enhanced the compressive strength, splitting tensile strength, surface abrasion and impact resistance of cementitious materials [13]. Addition of silica fume in fiber cementitious materials would improve the concrete properties and durability [13] and is beneficial for fiber dispersion [14,15]. In addition, silica fume would increase the bond between fibers and mortar [16] and strengthen the interfacial zone by decreasing the number and size of cracks [17]. In the fiber reinforced composites field, many previous researchers have studied the effect on the mechanical properties of cementitious materials with silica fume and steel fibers; however, few studies have evaluated the influence of material parameters on the mechanical properties of FCM especially for using parametric correlation analysis and nonlinear regression analysis. In addition, the study focused on the suitability of using the designed direct tensile testing method for FCM is also an innovation method to solve the possibly real tensile strength.

This study was aimed to evaluate the amount of silica fume addition and volume fraction of steel fiber on the engineering properties of cementitious materials. The experimental results were collected to carry out the analysis of variance to realize the relevancy and significance between material parameters and mechanical properties using Pearson correlation analysis. 

## 2. Experimental Program

### 2.1. Materials and Mix Proportion

Type I Portland cement conforming to ASTM C150-12 was used in all mixes. Silica fume with specific gravity of 2.20 and surface area of 22,500 m^2^/kg was used. The chemical components of cement and silica fume are shown in Table 1. The particle size of silica fume was about 0.1–0.2 μm. Silica fume (5% and 10% by weight of cement) was added to partially replace cement. Steel fiber used in this study was hooked-end bundle fiber with aspect ratios (*l*/d) of 40. The average length and tensile strength of steel fiber was 30 mm and 1100 N/mm^2^, respectively. Four volume fractions (*V_f_* = 0, 0.5, 1.0 and 2.0 vol.%) were used in each mix.

**Table 1:** Chemical components of cement and silica fume.

Chemical Composition (%)	SiO_2_	Al_2_O_3_	Fe_2_O_3_	CaO	MgO	SO_3_	L.O.I	K_2_O+Na_2_O (equivalent)	Others
Cement	21.2	5.4	3.2	63.8	2.0	2.2	0.7	0.8	0.7
Silica fume	91.5	0.2	0.7	0.4	1.5	0.5	1.4	1.9	1.9

The water/cement ratio (w/c) of 0.35 and 0.55 are selected for the reference mixes. The mix proportions of the control specimens are given in Table 2 and the targeted compression strength of mix A and mix B is 55 MPa and 35 MPa, respectively. The maximum size of coarse aggregate was 13 mm and the fineness modulus of fine aggregate was 2.87. Mixture slump was around 150 mm by using a high-range water-reducing admixture in the mixes. In this study, medium carbon steel was used for #6 rebar when the designed direct tensile strength testing.

**Table 2:** Mix design (kg/m^3^).

Mix no.	w/c	Water	Cement	Fine Aggregate	Coarse Aggregate	Superplasticizer
A	0.35	189.4	558.0	908.0	700.0	5.6
B	0.55	217.0	395.0	908.0	780.0	0

The coding used to identify “Mix No.” in column one of Table 1 should read: “A” and “B”, to represent the w/c of 0.35 and 0.55; “5” and “10”, to represent the dosages of silica fume at 5% and 10%; “f1”, “f2” and “f3”, to represent the volume fractions of steel fiber at 0.5%, 1.0% and 2.0%. Mixture slump was around 150 mm by using a high-range water-reducing admixture which is polycarboxylate type performed in accordance with type G in ASTM C494-13.

### 2.2. Specimens

Specimens with a total of 24 different mixes were cast. For each mix, total thirty specimens per series were tested included eighteen ψ100 mm × 200 mm cylindrical specimens for compressive strength test, six ψ150 mm × 300 mm cylindrical specimens for direct and splitting tensile strength test and six ψ150 mm × 64 mm cylindrical specimens for surface abrasion test and drop weight test. In addition, all the specimens were cured in saturated lime water until testing in accordance with ASTM C192-14.

### 2.3. Test Methods

The compressive strength test was performed in accordance with ASTM C39-14 and the splitting tensile strength test was conducted in accordance with ASTM C496-11. Also, the surface abrasion test was conducted following the specifications of ASTM C418-12. The surface abrasion test method covers the determination of abrasion resistance characteristics of FCM by subjecting the specimen to the impingement of air-driven silica sand. Weight loss and abrasion coefficient were determined accordingly.

**Figure 1:**
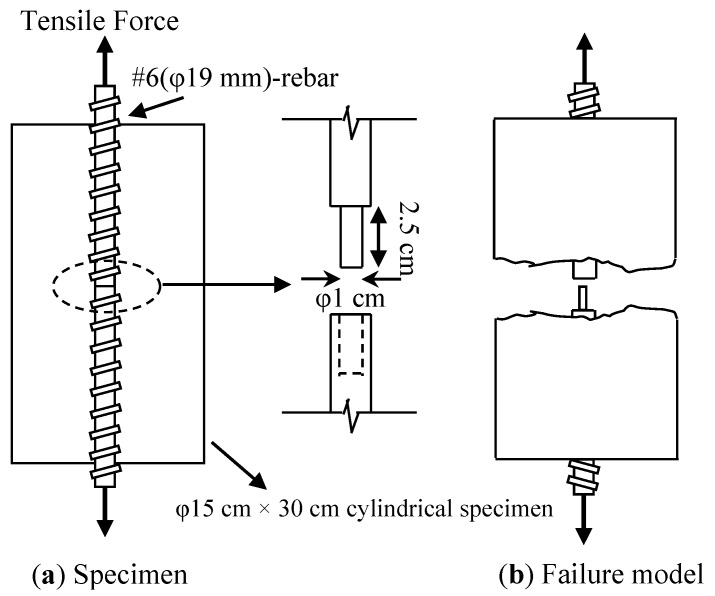
Schematic description of direct tensile testing (**a**) Specimen; (**b**) Failure model.

The designed direct tensile testing method is a relatively new test that is a modified version of those in previous studies [18,19] as shown in Figure 1. Two lengths of rebar (#6) were placed along the longitudinal axis of the ψ150 mm × 300 mm cylindrical specimen. A 10 mm diameter hole was drilled to a depth of 25 mm in the center of one rebar; the other rebar was machined into a ψ10 mm cylindrical plug at one end. To test the tensile strength, the exposed sections of the rebar were subjected to a load of 1 mm/min until failure. With the exception of compressive strength tests, all specimens were maintained in a curing room until the age of 120 days prior to testing.

The impact resistance was performed at the age of 150 days following the recommendation of ACI committee 544. It is suggested to repeat to drop a 63.5 mm-diameter and 4.54 kg steel ball from a height of 914 mm on the specimen until the first visible crack is found and then to record the drop number. Failure is defined as the crack opening to let specimen touch at least three of the four positioning lugs on the base plate.

## 3. Results and Discussion

### 3.1. Strength Properties

**Figure 2:**
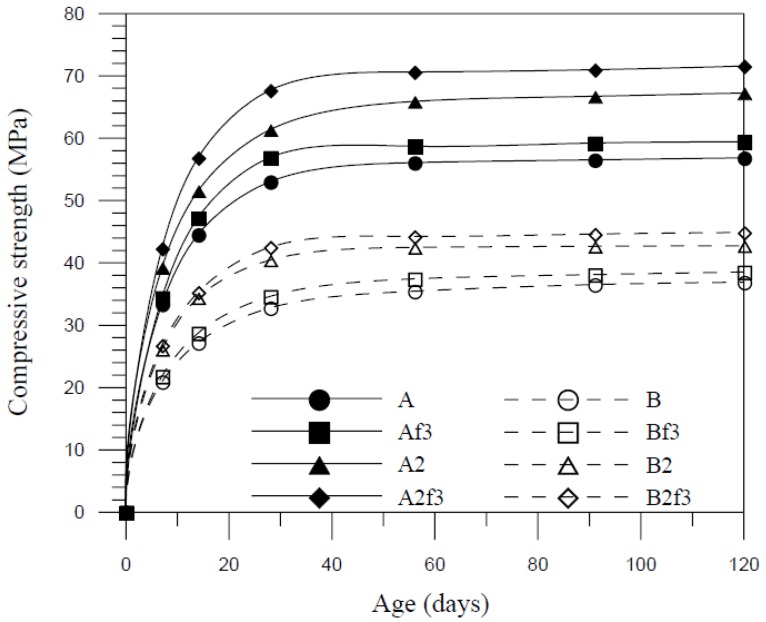
Compressive strength development curves.

The compressive strength development curves are plotted in Figure 2 for mix A and mix B with silica fume (10%) and fiber (2%). The incorporation of silica fume and silica fume mixed with fiber increased the average compressive strength by 16% and 22%, respectively. This research will focus on the mechanical property test that was carried out when the age of concrete reached 120 days and the strength change of concrete may not be considered (120-day strength is about the same as 56-day strength). The strength test results included compressive strength, splitting strength and direct tensile strength at the age of 120 days are summarized in Table 3.

**Table 3:** Strength results of fiber cementitious materials (FCM) specimens (MPa).

Mix no.	Compressive Strength	Splitting Strength	Direct Tensile Strength
A	56.85	3.86	3.51
Af1	57.66	4.20	3.73
Af2	60.18	5.44	4.10
Af3	59.43	5.51	4.12
A1	61.99	3.94	3.60
A1f1	62.71	4.23	3.92
A1f2	63.03	5.58	4.52
A1f3	63.62	6.29	4.61
A2	67.24	3.95	3.62
A2f1	68.21	4.44	4.04
A2f2	72.32	5.82	4.66
A2f3	72.77	6.55	4.71
B	36.90	3.11	3.26
Bf1	38.37	3.35	3.60
Bf2	38.96	3.58	3.69
Bf3	38.55	3.94	3.77
B1	39.83	3.38	3.49
B1f1	41.09	4.02	3.63
B1f2	41.97	4.57	3.78
B1f3	42.13	5.23	3.91
B2	42.48	3.65	3.51
B2f1	44.89	4.11	3.66
B2f2	45.32	4.77	3.83
B2f3	45.76	5.46	4.00

For compressive strength, the inclusion of silica fume significantly increased the compressive strength. The strengthening of mechanical properties is due to the pozzolanic activity of silica fume causing improved strength of the cement paste, the increased density of cementitious materials resulting from the fineness of silica fume and the consequent efficient reaction to form hydration products which filled the capillaries between cement and aggregate and the refined pore structure. For all the w/c ratios, 10% replacement of cement by silica fume considerably improved the compressive strength with respect to control specimens. The addition of steel fiber had a small effect on the compressive strength. It indicated that the compressive strength increased 7% to 9% with every 5% replacement silica fume added. In terms of the volume fraction and aspect ratio of fibers, the strength enhanced 1% to 2% with increasing of the steel fiber addition and the aspect ratio in FCM.

Although the addition of silica fume had no significant influence to splitting strength in low w/c (+2% for 10% silica fume addition), the splitting strength increased with the increasing of silica fume in high w/c (+17% for 10% silica fume addition). It is due to that silica fume addition to the cementitious materials improved and enhanced the microstructures. It was more effectively in high w/c materials with larger porestructures which can be fill by the incorporation of silica fume. In terms of dispersion, the silica fume was used to help the fiber dispersion. The silica fume can affect the fiber pullout resistance of mechanical interlock between fiber and matrix, and can be expected to influence composite performance to some extent regardless of fiber type and aspect ratios. The table illustrated that the splitting strength heightened with the increase of the added amount of fiber, but the splitting strength was found to decrease considerably at 2% fiber volume. This is because of the poor fiber dispersion leaded to the debonding and pullout at the interface between fiber and cementitious. The addition of 5% replacement silica fume can improve the interfacial bond between fibers and cementitious.

Among the existing test methods, it is believe that the direct tensile strength test is the best in producing realistic measurement of the uniaxial tensile strength of cementitious materials. From these results, it may be seen that the direct tensile strength was considerably influenced by the dispersion of fibers depended on the dosages of silica fume. The results indicated that good fiber dispersion can increase the tensile strength about 20% to 30%. The capacity for resisted direct tension of FCM depends on the interfacial bond between fiber and matrix. Even more fibers benefit greatly upon transferring energy and restraining crack. In conclusion, the addition of silica fume and steel fiber can increase the ability of crack-arresting. The optimum addition for compressive, splitting and direct tensile strength of mix A and B is the mixture with 5% replacement silica fume and 2% fiber volume eliminated from the 10% replacement silica fume due to the cost and benefit consideration.

### 3.2. Abrasion Resistance

**Figure 3:**
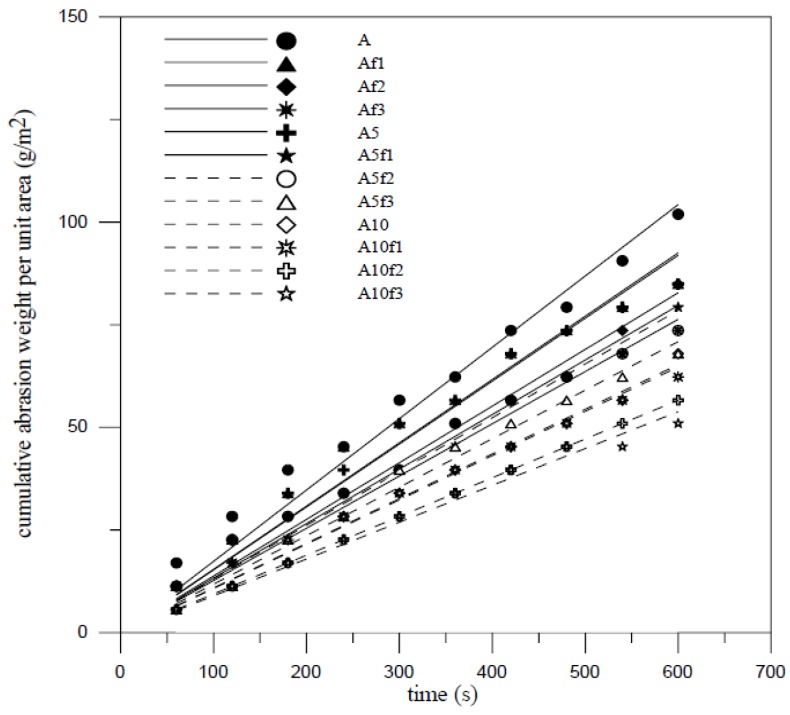
Cumulative abrasion weight per unit area with mix A.

**Figure 4:**
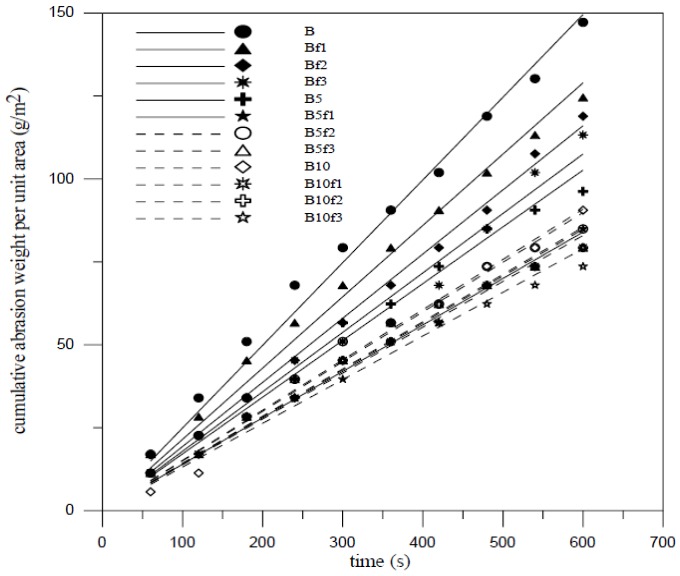
Cumulative abrasion weight per unit area with mix B.

**Figure 5:**
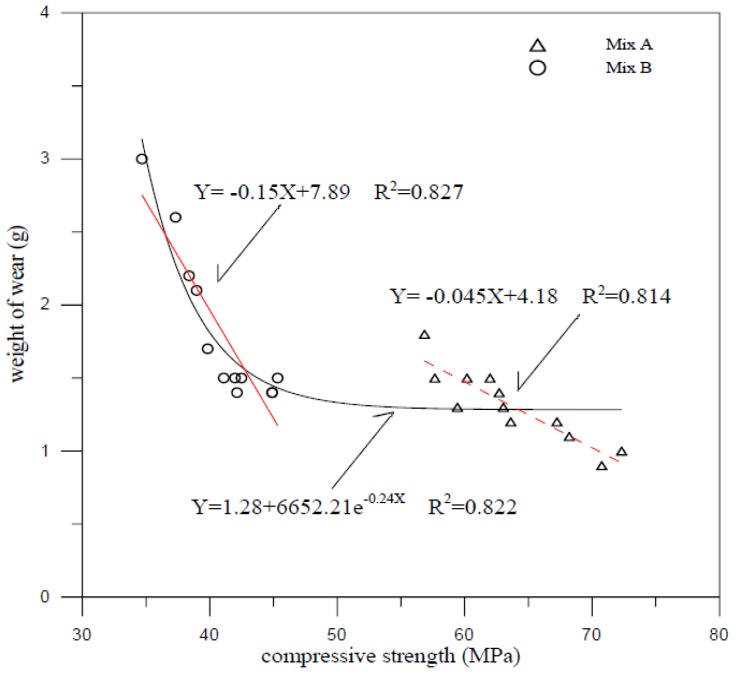
Abrasion resistance *versus* Compressive strength curves.

Surface abrasion resistance was determined according ASTM C418 standard and performed for a period of 60 second. Although surface abrasion has great influence on the addition of silica fume and fiber volume, the compressive strength is one of the most important factors responsible for the abrasion resistance of FCM [20]. Abrasivity defines the ability of FCM to abrasion resistance. The abrasivity can be determined by A-t curve using linear regression fit method and the A-t formula presents as following:

*A* = *Kt* (1)

where *A* is cumulative surface abrasion mass per unit area (g/m^2^); *t* is performed time (s); and *K* is abrasivity (g/m^2^-s). Linear regression analysis is applied to obtain the relationship between cumulative surface abrasion for 40 aspect ratio and time and is shown in Figure 3 and Figure 4. The ability of FCM to abrasion resistance increases by the additions of silica fume and fiber added. The compactness of materials surfaces by silica fume addition and the inhibition of crack and spalling by fiber addition were useful to enhance the abrasion resistance as well as interactions between moving particles and concrete surfaces. To raise the additions, the abrasivity may decrease. The abrasivity is 0.150 g/m^2^-s, 0.232 g/m^2^-s and 0.122 g/m^2^-s for mix A, B and A with 2% fiber volume added, respectively as indicated in Figure 3 and Figure 4. All abrasion test results show that the compressive strength was an important factor affecting the abrasion resistance of FCM. Figure 5 shows that the weight of wear decreased linearly as compressive strength increased. From the whole viewpoint of compressive strength, the weight of wear seems saturated with little growth in high compressive strength.

### 3.3. Drop Weight Test

**Figure 6:**
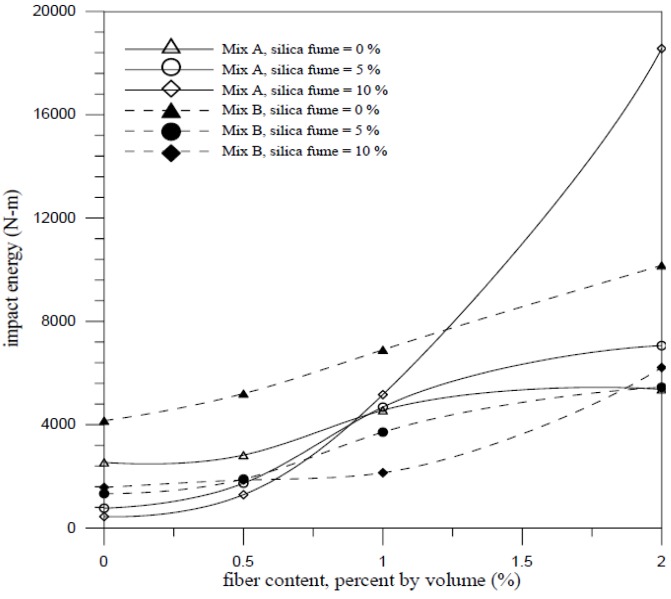
Relationship between impact energy and fiber content.

Impact energy can be determined by the formula as following:

*W* = *Nmgh* (2)

where *W* is impact energy (N-m); *m* is weight of steel ball (kg); *g* is gravitational acceleration (m/s^2^); *h* is drop distance (m); and *N* is rupture impact number. The relationship between impact energy and fiber content is shown in Figure 6, which indicates the specimen with higher w/c has greater increase in energy absorbing capacity than the specimen with lower w/c. The impact energy of high w/c increased 65% than that of low w/c due to the brittle. The presence of silica fume rendered the concrete more brittle due to the higher strength properties as mentioned previously. For fiber cementitious materials, higher fiber contents were needed to maintain the ductility and the impact energy increased with the increased of the composite of silica fume and steel fiber added.

**Figure 7:**
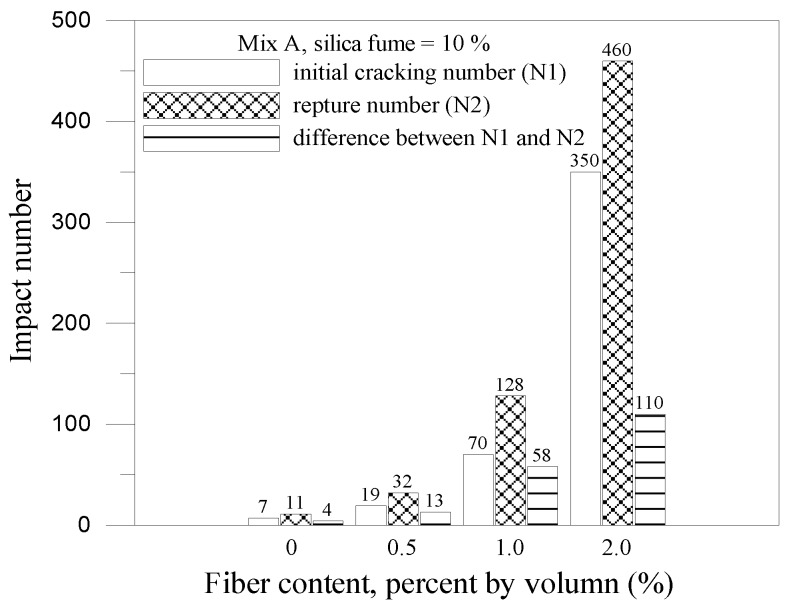
Impact results for mix A with 10% silica fume.

**Figure 8:**
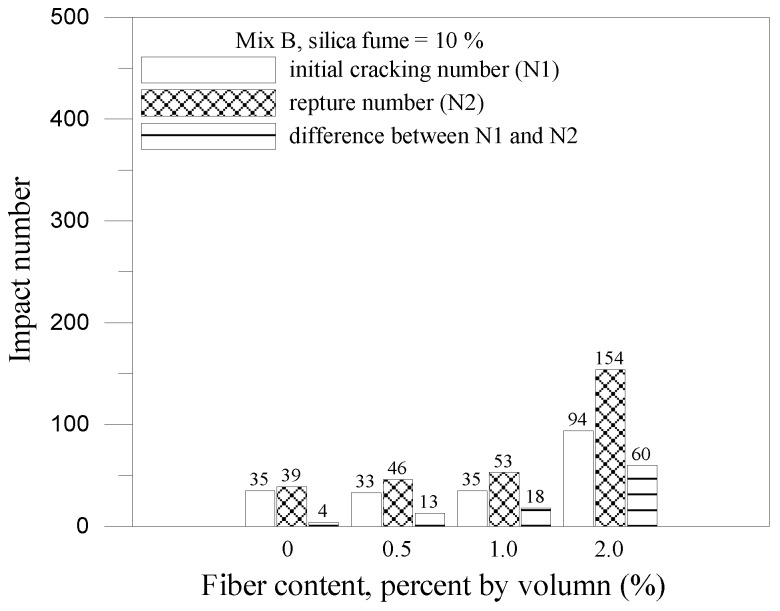
Impact results for mix B with 10% silica fume.

**Figure 9:**
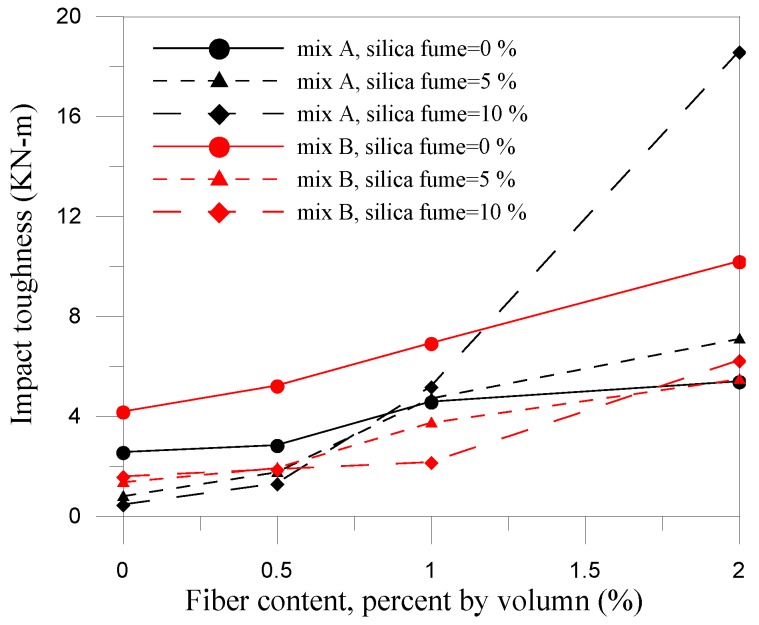
Impact toughness *versus* fiber content curves.

**Figure 10:**
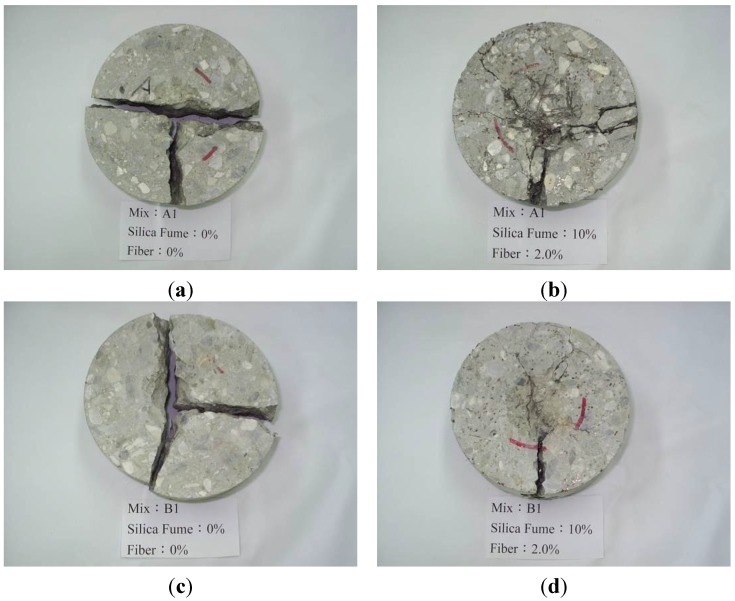
Part of the FCM specimens after ultimate failure (**a**) A specimen; (**b**) A2f3 specimen; (**c**) B specimen; (**d**) B2f3 specimen.

The impact results for mix A and mix B with 10% silica fume are shown in Figure 7 and Figure 8, respectively. With the addition of silica fume and fiber volume increased, the initial cracking impact number, impact rupture number, different between initial and rupture and the impact energy raised obviously. The composite for mix A with 10% silica fume and 2% fiber volume had the best optimum ability of impact resistance and the impact energy can be raised 7.3 times than the plain concrete. However, the composite for mix B with 10% silica fume and 2% fiber volume only had an increase up to 50% than the control specimens (B specimens). Silica fume improved the microstructure, eliminated voids contents in the composites and enhanced the interface bonding between fiber and matrix. It indicated that the composite had a great ability to absorb kinetic energy and arrest crack due to the combined effect of the steel fiber and silica fume. The relationship between impact energy and fiber content for all mixes is illustrated in Figure 9. The fiber composites without silica fume had relatively higher impact energy than other FCM specimens. This is because the composites without silica fume had larger pore and voids, which provided energy dissipation. The failure patterns of the FCM specimens after impact ultimate failure are shown in Figure 10. Inclusion of fibers in composites can restrict cracks and resist parted composites effectively. In conclusion, fiber bridging can effectively inhibit crack formation and respite the rate of cracking under impact condition.

### 3.4. Parametric Correlation Analysis

The foregoing reports concluded that the presence of silica fume and steel fiber influenced the mechanical properties, but the individual effect and direction was needed to measure further by the correlation coefficient. Pearson correlation coefficient measured the relationship between mechanical properties included compressive strength (*f_c_’*), splitting strength (*f_sp_*), direct tensile strength (*f_dir_*), abrasion coefficient (*A_c_*) and impact number of ultimate failure (*N*) as well as material variables included silica fume content (*W_s_*) and steel fiber content (*V_f_*), and which is the most widely used type of correlation coefficient. Pearson coefficients of parametric correlations are tabulated in Table 4. The silica fume content was positively related to the compressive strength and negatively related to the abrasion resistance. Also, the fiber volume fraction was positively related to the splitting strength, direct tensile strength and impact resistance for each mix, which corresponded to the results in the above-mentioned articles. The beneficial effect of silica fume addition was more markedly on the compressive strength than that on the abrasion coefficient due to larger Pearson coefficient was observed. Nevertheless, the Pearson coefficient only measured the effect of a single variable on mechanical properties. The interaction between each variable on mechanical properties required some further explanations by analysis of variable. 

**Table 4:** Pearson coefficients of parametric correlations.

Paired sample	w/c	Pearson correlation (r)	Significant (2-tailed)
*f_c_’ vs.* *W_s_*	0.35	0.94	0.00 **
	0.55	0.94	0.00 **
*f_c_’ vs.* *V_f_*	0.35	0.23	0.48
	0.55	0.26	0.42
*f_sp_ vs.* *W_s_*	0.35	0.19	0.55
	0.55	0.56	0.06
*f_sp_ vs.* *V_f_*	0.35	0.92	0.00 **
	0.55	0.76	0.00 **
*f_dir_ vs.* *W_s_*	0.35	0.38	0.22
	0.55	0.36	0.25
*f_dir_ vs.* *V_f_*	0.35	0.81	0.00 **
	0.55	0.87	0.00 **
*A_c_ vs.* *W_s_*	0.35	−0.80	0.00 **
	0.55	−0.86	0.00 **
*A_c_ vs.* *V_f_*	0.35	−0.53	0.08
	0.55	−0.29	0.36
*N vs.* *W_s_*	0.35	0.22	0.49
	0.55	−0.58	0.05 *
*N vs.* *V_f_*	0.35	0.75	0.01 **
	0.55	0.73	0.01 **

Note: * Correlation is significant at the 0.05 level (2-tailed); ** Correlation is significant at the 0.01 level (2-tailed).

## 4. Conclusions

The cementitious materials containing steel fiber showed an increase in compressive and tensile strength with increasing the amount of fiber volume fraction from 0.5% to 2.0%, unless the fiber volume was so high that the poor fiber dispersion tended to have a negative effect on strength properties, especially the compressive strength. The pozzolanic effect of silica fume was used to help the fiber dispersion and improve the interfacial bond between fibers and cementitious. The fiber cementitious materials with high strain capacity and toughness can reflect the higher ability of crack-arresting. The optimum composite for compressive and tensile strength is the mixture with 5 wt.% replacement silica fume and 2 vol.% fiber volume. ASTM C418 is successful in evaluating the surface abrasion resistance of fiber cementitious materials. The surface abrasion resistance increased as the w/c content decreased, the replacement silica fume increased and the fiber volume increased. Good linear correlations existed between weight of wear and compressive strength by linear regression analysis. When 10% replacement silica fume and 2% fiber volume were both incorporated into the cementitious materials, the surface abrasion and impact resistance performance can be enhanced considerably. In addition, Pearson correlation coefficient was conducted to evaluate the influence of the material variables, which was corresponding to the experiment result.
